# The Influence of Sex and Fly Species on the Development of Trypanosomes in Tsetse Flies

**DOI:** 10.1371/journal.pntd.0001515

**Published:** 2012-02-14

**Authors:** Lori Peacock, Vanessa Ferris, Mick Bailey, Wendy Gibson

**Affiliations:** 1 School of Biological Sciences, University of Bristol, Bristol, United Kingdom; 2 School of Clinical Veterinary Science, University of Bristol, Bristol, United Kingdom; Lancaster University, United Kingdom

## Abstract

Unlike other dipteran disease vectors, tsetse flies of both sexes feed on blood and transmit pathogenic African trypanosomes. During transmission, *Trypanosoma brucei* undergoes a complex cycle of proliferation and development inside the tsetse vector, culminating in production of infective forms in the saliva. The insect manifests robust immune defences throughout the alimentary tract, which eliminate many trypanosome infections. Previous work has shown that fly sex influences susceptibility to trypanosome infection as males show higher rates of salivary gland (SG) infection with *T. brucei* than females. To investigate sex-linked differences in the progression of infection, we compared midgut (MG), proventriculus, foregut and SG infections in male and female *Glossina morsitans morsitans*. Initially, infections developed in the same way in both sexes: no difference was observed in numbers of MG or proventriculus infections, or in the number and type of developmental forms produced. Female flies tended to produce foregut migratory forms later than males, but this had no detectable impact on the number of SG infections. The sex difference was not apparent until the final stage of SG invasion and colonisation, showing that the SG environment differs between male and female flies. Comparison of *G. m. morsitans* with *G. pallidipes* showed a similar, though less pronounced, sex difference in susceptibility, but additionally revealed very different levels of trypanosome resistance in the MG and SG. While *G. pallidipes* was more refractory to MG infection, a very high proportion of MG infections led to SG infection in both sexes. It appears that the two fly species use different strategies to block trypanosome infection: *G. pallidipes* heavily defends against initial establishment in the MG, while *G. m. morsitans* has additional measures to prevent trypanosomes colonising the SG, particularly in female flies. We conclude that the tsetse-trypanosome interface works differently in *G. m. morsitans* and *G. pallidipes*.

## Author Summary

In tropical Africa human and livestock diseases caused by parasitic trypanosomes are transmitted by bloodsucking tsetse flies. In the fly, trypanosomes undergo a complex cycle of proliferation and development during their remarkable journey from the midgut to the salivary glands. At every step of the way, the flies mount robust immune defences against trypanosome infection and consequently most flies fail to develop a transmissible infection. Previous work has shown a sex difference in the numbers of salivary gland infections with *Trypanosoma brucei*: male flies are more susceptible to salivary gland infection than females. Here we explored possible reasons for this. Infections developed in the same way in both male and female flies until the final stage of salivary gland invasion and colonisation. We conclude that the salivary gland environment in the female fly is much more inhospitable for trypanosomes, perhaps because of a greater immune response. Comparison of two different tsetse species showed very different levels of trypanosome resistance in the midgut and salivary glands.

## Introduction

During transmission of a pathogen, selection in the invertebrate vector may be of profound importance in dictating which pathogen genotypes are most prevalent in mammalian hosts. This evolutionary pressure can select for particular combinations of pathogen and vector species, and weed out less fit pathogen phenotypes regardless of any competitive advantage in the mammalian host, such as virulence or drug resistance.

Tsetse flies (Diptera: Glossinidae) serve as vectors of several pathogenic trypanosome species in subsaharan Africa, but typically manifest high levels of resistance to infection [1], [2]. Resistance mechanisms operate at a number of levels and time points during the trypanosome's complex developmental cycle within the fly. For *Trypanosoma brucei*, trypanosomes first establish infection in the tsetse midgut (MG), initially in the gut lumen with subsequent invasion of the ectoperitrophic space via the peritrophic matrix (PM) enclosing the bloodmeal. The antimicrobial defences operating in the MG, such as antimicrobial peptides, lectins and reactive oxygen intermediates [3], [4], [5], [6], [7], ensure that a high proportion of infections are cleared at this early stage. In the laboratory, these defences can be counteracted by, for example, feeding the flies on lectin-binding sugars or anti-oxidants [8], [9], [10] or knocking down expression of specific antimicrobial peptides or proteins using RNA interference [4], [11].

Of the MG infections that persist, few subsequently result in a salivary gland (SG) infection and it is evident that the trypanosomes experience a severe population bottleneck, as the SG are invaded and colonised by very small numbers of trypanosomes [12], [13]. The barriers to SG infection are unknown, but there are several points along this complex pathway where progression may potentially be blocked. From the MG, trypanosomes move anteriorly to invade the proventriculus and penetrate through the PM before migrating to the SG via the foregut. The proventriculus is known to be a highly immunogenic tissue [5] and this could influence the success of trypanosome invasion of the foregut or the differentiation from MG procyclics to migratory forms. Escape of trypanosomes from the proventriculus into the foregut would also be blocked if trypanosomes were unable to penetrate the PM. Little is known of SG immune responses, but these are also likely to be vigorous judging by the frequent failure of migratory trypanosomes to colonise the SG and establish infection [2], [12], [13]. A recent survey of genes expressed in tsetse SG revealed a large variety of potential immunity-related molecules, some of which are also expressed by MG and fat body tissues [14].

Unlike other dipteran vectors such as mosquitoes, sand flies and black flies, both male and female tsetse feed on blood and hence serve as trypanosome vectors. Intriguingly, fly sex appears to influence susceptibility to trypanosome infection. Male flies (*Glossina morsitans morsitans*, *G. m. centralis, G. pallidipes, G. fuscipes fuscipes*) showed higher rates of SG infection with *T. brucei* than females [15], [16], [17], and it has been suggested that a sex-linked recessive gene is involved [16]. The underlying cause of this sex difference in susceptibility is not known.

To investigate sex-linked differences in the development of trypanosome infections in tsetse, we compared the development of MG, proventriculus, foregut and SG infections in male and female flies of *G. m. morsitans*. Infections developed in the same way in both male and female flies until the final stage of SG invasion and colonisation, when the SG environment in the female fly proved to be much more inhospitable for trypanosomes. Comparison of *G. m. morsitans* with another tsetse species, *G. pallidipes*, showed a similar sex difference in susceptibility to SG infection though less pronounced. However, *G. pallidipes* manifested much greater resistance to MG infection than *G. m. morsitans* and remarkably little resistance to SG infection. It thus appears that these two tsetse species have evolved very different strategies to counter trypanosome infection.

## Results

### *Glossina morsitans morsitans*

**Figure 1:**
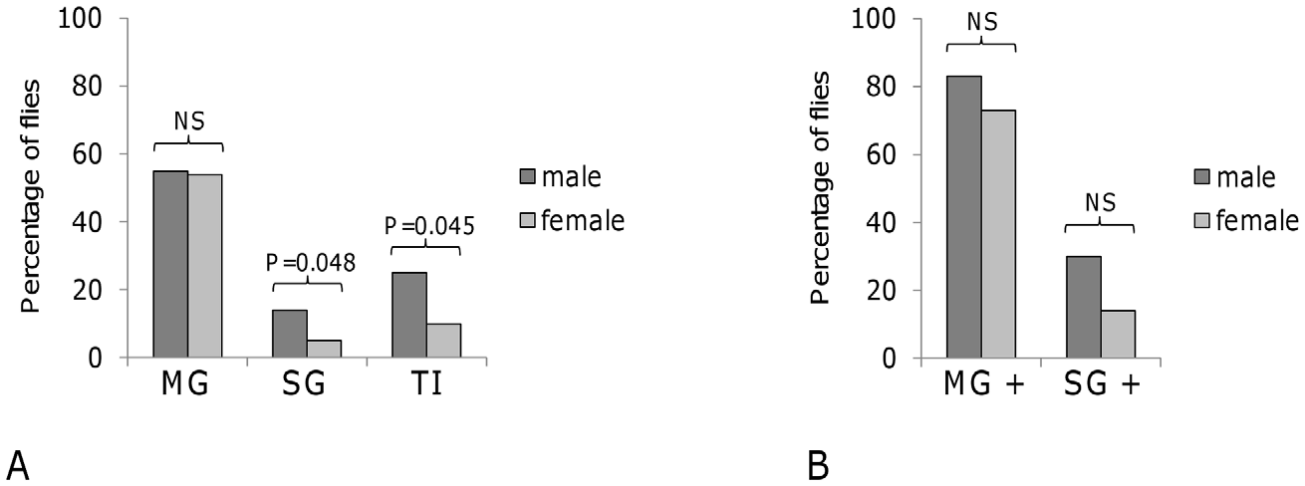
Sex differences in infection rates with *T. b. brucei* in *G. m. morsitans*. Individually-caged male and female flies were fed an infected feed supplemented with N-acetyl-glucosamine; data combined from two replicate experiments. Spit samples were collected between days 7 to 28, and flies were dissected to score midgut (MG) and salivary gland (SG) infections on day 28. Transmission index (TI) is the percentage of flies with MG infection that also developed SG infection. P values from chi-squared analysis to compare differences between male and female flies; NS = not significant. **A.** Infection rates at dissection. N = 87 males, 95 females. **B.** Analysis of positive spit samples produced by 40 male and 37 female flies. Left: Percentage of MG infections that produced a positive spit sample. Right: Percentage of positive spit samples that resulted in SG infection.

Comparison of male and female *G. m. morsitans* infected with *T. b. brucei* J10 confirmed previous findings that male tsetse flies establish greater numbers of SG infections of *T. brucei* than females [15], [16], [17]. While there was no significant difference in MG infection rates, a significantly higher proportion of MG infections progressed to SG infection in male than in female flies. The transmission index (TI = infected salivary glands/infected MGs) for male flies was over twice that for female flies (*P* = 0.045; Fig. 1A).

The progression of infection in these flies was monitored at points of transition in the developmental cycle to investigate the nature of the barriers to SG infection and influence of fly sex. In established MG infections, the first event we recorded was invasion of the proventriculus by trypanosomes migrating anteriorly within the ectoperitrophic space. In flies dissected 10–14 days after infection, only about three quarters had an infected proventriculus and there was no difference in infection rates between males and females (Table 1). In the proventriculus, trypanosomes arrest in G2 before undergoing an asymmetric division that yields one short and one long epimastigote; these are migratory stages and the short epimastigote is believed to invade the SG [18], [19], [20]. Asymmetric dividers were found in about 75% of infected proventriculi, with no significant difference between male and female flies (Table 1).

**Table 1:** Proventriculus infection rates of *T. b. brucei* J10 in *G. m. morsitans*.

*Glossina morsitans morsitans*	Male	Female
Number of flies dissected	32	32
Infected proventriculus	24/32 (75%)	25/32 (78%)
Proportion of infected proventriculi containing asymmetric dividers	18/24 (75%)	19/25 (76%)

All flies had an infected midgut; infected feed was supplemented with N-acetyl-glucosamine.

The next event is that the migratory trypanosomes invade the foregut and can be found in the salivary exudate or spit, a mixture of regurgitated foregut contents and saliva from the SG produced by flies when they probe a surface with the proboscis [18], [21]. To examine the foregut contents, we used individually-caged flies, which were allowed to probe onto warm microscope slides 7–28 days after infection; dissection results for these flies at day 28 are shown in Fig. 1A. Trypanosome-positive spit samples were only obtained from those flies subsequently found to have MG infection, but some flies (about 20%) with MG infection did not produce a positive spit sample during the whole observation period (Fig. 1B). This reflects the failure of about 40% (27 of 64) MG infections to infect the proventriculus and produce asymmetric dividers (Table 1). Only a small proportion of spit-positive flies finally developed SG infection, just over 20% combining males and females (Fig. 1B), which means that in the majority of infections the migratory trypanosomes either failed to reach the SG or to colonise them. Attrition was greater in female than male flies (Fig. 1B), although the sex difference was not statistically significant.

**Figure 2:**
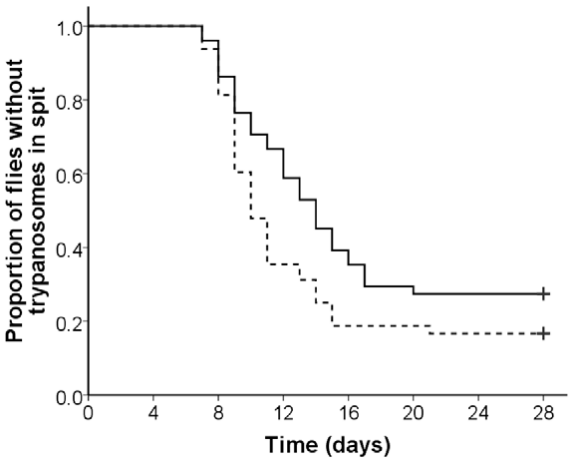
Sex difference in first appearance of trypanosomes in spit samples. Comparison of individually caged male (dotted line) and female (black line) *G. m. morsitans* infected with *T. b. brucei* J10; infected feed was supplemented with N-acetyl-glucosamine. The data from two replicate experiments, each including male and female flies are displayed as a Kaplan Meier survival plot (males: n = 40; females: n = 37). Vertical ticks indicate points after which flies were not sampled in experiments 1 (day 18) and 2 (day 28) (censored data). Female flies were significantly slower in producing trypanosome positive spit samples than males (*P*<0.05).

The relative proportions of trypanosome developmental stages in individual stained spit samples from male and female flies were similar (Table 2); at this early stage of infection, few metacyclics were present. Additionally, the trypanosome composition of the spit sample had no bearing on whether a fly subsequently developed SG infection, as there was no significant difference in numbers of developmental stages in spit samples from flies with or without SG infection when dissected at 28 days (Table 2). However, there was a significant effect of gender on the rate at which flies became spit-positive, the females lagging behind the males (Fig. 2: median time to positivity 10 or 14 days for males or females, respectively, *P*<0.05). It was noticed that often very few trypanosomes (typically <5) were present in spit samples from flies that became positive on or after 12 days, the majority of which were female. It is possible that colonisation is adversely affected by the later arrival and smaller numbers of migratory trypanosomes in female compared to male flies. However, this hypothesis was not borne out by statistical analysis of the combined data from male and female flies: of 48 flies that gave their first positive spit sample early (7–11 days after infection), 9 had positive SG at dissection (19%), whereas of 29 flies that produced their first positive sample late (12–21 days after infection), 6 were SG positive at dissection (21%) (*P* = 1.00). So time of migration to the SG does not affect the success of SG colonisation.

**Table 2:** Breakdown of trypanosome developmental stages in spit samples.

Category	Subcategory	N	Long trypomastigotes	Asymmetric dividers	Short & long epimastigotes	Metacyclics
Fly sex	Male	24	7.07±0.85	6.93±0.74	2.90±0.53	1.82±0.31
	Female	24	6.05±1.24	4.95±1.09	2.71±0.78	0
Salivary glands	Infected	12	6.81±1.34	5.17±1.18	2.45±0.84	1.82±0.48
	Uninfected	36	6.30±0.68	6.71±0.60	3.16±0.43	0

Results for 48 spit samples collected from *G. m. morsitans* infected with *T. b. brucei* J10 on days 8–18. Infected feed was supplemented with N-acetyl-glucosamine. Values are the square-root transformed means ± standard errors for the number of trypanosomes of each cell type per spit sample.

### *Glossina pallidipes*

We compared infection rates of *G. m. morsitans* with those of *G. pallidipes* using the same strain of *T. b. brucei*, J10. Without immunosuppressive supplements, MG infection rates were very low in *G. pallidipes* compared to *G. m. morsitans* (Table 3). The addition of N-acetyl-glucosamine (NAG) or L-glutathione (GSH) to the infected feed has been shown to enhance MG infection rates [8], [9] in *G. m. morsitans* by blocking antimicrobial lectins or reactive oxygen species respectively, and this is also evident from the data collected here for *G. m. morsitans* (Table 3); there is no effect of these supplements on SG infection rates except as a result of increased numbers of MG infections [8], [22]. However, in contrast to *G. m. morsitans*, NAG appeared to be totally ineffective in boosting numbers of MG infections in *G. pallidipes*: no infected MG were found with NAG in *G. pallidipes* compared with 54.4% infected MG in *G. m. morsitans* (Table 3). The addition of GSH resulted in a large increase in numbers of infected MG for *G. pallidipes* (50.0% with GSH versus 1.3% without GSH), similar to the effect seen in *G. m. morsitans* (81.0% with GSH, 11.3% without GSH), but significantly lower comparing the two fly species (*P* = 0.018) (Table 3).

**Table 3:** Effect of supplements on infection rates.

	Infected midguts		Transmission index (TI)	
Supplement	Gmm	Gp	Gmm	Gp
Control	8/71 = 11.3%	1/78 = 1.3%	1/8 = 12.5%	1/1 = 100%
NAG	99/182 = 54.4%	0/53 = 0%	17/99 = 17.2%	0/0 = 0%
GSH	77/95 = 81.0%	34/68 = 50.0%	3/77 = 3.9%	30/34 = 88.2%

Comparison of infection rates of *G. m. morsitans* (Gmm) and *G. pallidipes* (Gp) fed *T. b. brucei* J10 in horse blood (control) or horse blood supplemented with N-acetyl-glucosamine (NAG) or glutathione (GSH). Pooled results from male and female flies and replicate experiments.

**Figure 3:**
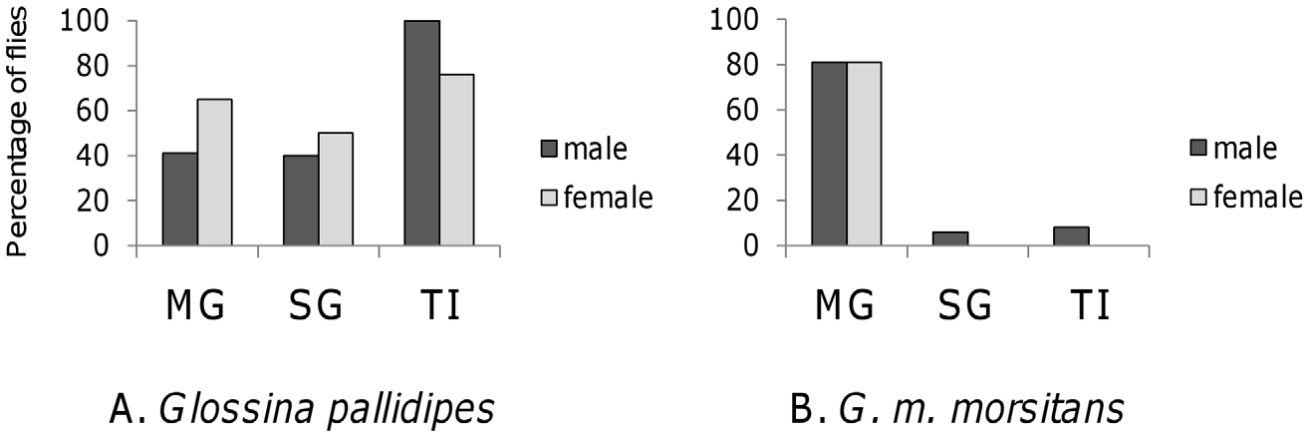
Sex differences in *G. pallidipes* and *G. m. morsitans* infected with *T. b. brucei*. Infected feeds were supplemented with glutathione. A. *G. pallidipes* N = 42 males, 26 females. B. *G. m. morsitans* 47 males, 48 females. Differences between MG, SG and TI results for male and female *G. pallidipes* were not significant by chi-squared analysis. No female *G. m. morsitans* had infected SG in this experiment. Pooled data from male and female flies in Table 3.

The high MG infection rates obtained with GSH enabled us to examine SG infection rates in *G. pallidipes* (Fig. 3). Comparison with *G. m. morsitans* showed that transmission was far more efficient in *G. pallidipes*, despite lower MG infection rates; the SG infection rates and TI for both sexes of *G. pallidipes* were significantly higher than for *G. m. morsitans* (*P*<0.0001; Fig. 3). In fact, all 17 male *G. pallidipes* with MG infection also had infected SG (TI = 100%) compared to only three of 38 male *G. m. morsitans*, and 13 of 17 female *G. pallidipes* with MG infection also had infected SG whereas none of 48 female *G. m. morsitans* with infected MG had infected SG. As for *G. m. morsitans*, there was a sex difference in TI and *G. pallidipes* males had a higher TI than females, though this was not significant. Although female *G. pallidipes* had higher MG and SG infection rates than males, the differences were not significant either (Fig. 3).

### Salivary gland hypertrophy

The *G. pallidipes* colony from which our experimental flies were derived suffers from infection with a virus that causes the SG to become much enlarged, a condition called salivary gland hypertrophy (SGH) [23], [24]. Although the prevalence of SGH is relatively low in the colony (3.8%), PCR diagnosis indicates that almost all flies are infected with SGH virus [23]. Viral load is significantly higher in symptomatic flies [24], suggesting that while most flies control viral infection and are asymptomatic, a minority succumb and develop SGH.

As it is not known how SGH affects trypanosome infection, we analysed whether there was an association between trypanosome infection and SGH in *G. pallidipes* infected with *T. b. brucei* (J10 and other strains) dissected at 28 days or later. The observed prevalence of SGH in our experimental flies at dissection was 11% (43 of 402). Of the 43 flies with SGH, 38 had infected SG (88%), a significantly greater level of infection than flies with normal SG (247 SG infected of 359 flies, 69%; *P* = 0.008). Although SGH is positively correlated with trypanosome infection, the large number of SG positive flies without SGH (69%) shows that SGH is by no means essential for SG colonisation by trypanosomes in *G. pallidipes*.

## Discussion

The tsetse fly is unusual among dipteran vectors of disease because both sexes feed on blood and hence transmit pathogenic trypanosomes. However, the sexes are not equally efficient vectors and males have been found to be more susceptible to infection with *T. brucei* than females [15], [16], [17]. To explore the underlying basis of this sex difference, we compared infections in male and female *G. m. morsitans* at a number of points in the trypanosome's developmental cycle within the alimentary tract and SG of the fly. Levels of attrition were similar in both male and female flies, until the final stage of SG invasion and colonisation. The only difference detected was among the trypanosomes that migrate from the MG to the SG via the foregut: in female flies these appeared later than in males. However, there was no detectable difference in the success of early or late migrating populations in invading and colonising the SG, so differential attrition at the trypanosome-SG interface remains the only underlying explanation for the observed sex difference.

It appears that migratory trypanosomes encounter a very hostile environment in the SG. In *G. m. morsitans* SG colonisation was frequently unsuccessful and only about 20% of flies positive for migratory trypanosomes in spit samples were subsequently found to have SG infection. Compared to the MG, little is known about the functional immune response of the SG to trypanosomes, but there are now detailed studies of the SG transcriptome and proteome that describe an armoury of potential antimicrobial defensive molecules [14], [25]. Presumably the host-parasite interaction in the SG is intensified by the invasive nature of trypanosome attachment, as the epimastigotes form extensive cell-cell junctions with the epithelial cells via the flagellar membrane [26]. We hypothesize that the SG environment of the female fly is far less hospitable than that found in the male fly, thus leading to lower rates of SG infection in female flies, but the factors accounting for this difference remain to be identified.

Why might this sex difference in resistance to trypanosome infection have arisen? In nature, because of the slow rate of tsetse reproduction, selection for female longevity must be intense. Each female fly gives birth to a fully grown larva every 8–9 days, the first larva being produced about 16 days after emergence and mating. In contrast, male flies reach sexual maturity within a week and can mate several times. For survival of the species, it is thus imperative that female tsetse live at least 24 days. Survival data from the field support this: in Zimbabwe the estimated mean ages of female *G. m. morsitans* and *G. pallidipes* were 29 and 48 days respectively, compared to about 15 days for males of both species [27]. Since *T. brucei* takes a minimum of about two weeks to complete its life cycle, female flies are more likely than males to be exposed to prolonged SG infection. If this is detrimental to fly survival, might trypanosomes themselves have driven the sex difference in resistance to SG infection? Few studies have addressed the impact of trypanosome infection on tsetse fitness. There was no effect of trypanosome MG infection on tsetse mortality, although fecundity of infected females decreased [28]. Tsetse with SG infection take longer to feed than uninfected flies and show altered composition of the saliva [29], implying that SG infection may indeed prejudice survival of flies in the wild.

We found differences in the host-parasite interaction in *G. pallidipes* compared to *G. m. morsitans* and there is a marked species difference in fly susceptibility to *T. b. brucei* infection. In both *G. m. morsitans* and *G. pallidipes* the immune responses of the MG are robust and capable of destroying most trypanosomes before they have a chance to establish infection. These defences can be mitigated by use of NAG or GSH in *G. m. morsitans*, but only GSH was effective in *G. pallidipes*. In contrast, whereas only a small proportion of MG infections result in SG infection in *G. m. morsitans*, the migration of trypanosomes from MG to SG seems to proceed without hindrance in *G. pallidipes*. A comparative study of the humoral immune response of these two species showed that *G. pallidipes* has a higher baseline level of attacin in the fat body, proventriculus and midgut than *G. m. morsitans*; in *G. pallidipes* attacin levels increased after blood feeding and knockdown of attacin expression by RNA interference increased susceptibility to trypanosome infection [30]. Higher attacin levels may therefore be the underlying cause of the low MG infection rates we observed in *G. pallidipes*. The fact that immunosuppression with either NAG or GSH failed to work as efficiently in *G. pallidipes* compared to *G. m. morsitans* indicates that lectins and reactive oxygen species play a greater part in MG defence in *G. m. morsitans*.

Despite its refractoriness to MG infection, we found that *G. pallidipes* was far more permissive than *G. m. morsitans* in allowing progression to SG infection, particularly in male flies. We also found a positive correlation between SG infection and viral SGH in *G. pallidipes*, suggesting the possibility that susceptibility to SG colonisation is associated with viral infection. This echoes the association of infection with the secondary endosymbiont *Sodalis glossinidius* with susceptibility to MG infection with trypanosomes [1], [31]. The *G. pallidipes* colony shows a high prevalence of SGH virus infection, though relatively few flies have frank SGH. Interestingly the prevalence of SGH is significantly higher in male than female flies [23]. Viral infection may lead to changes in the SG epithelium that favour trypanosome colonisation; alternatively, flies that succumb to viral infection and develop SGH may have lower levels of immunity in the SG. On the other hand, *G. pallidipes* may naturally manifest low levels of immune defence in the SG, explaining both its susceptibility to virus and trypanosomes. Similar arguments have been rehearsed for the interaction between *Sodalis glossinidius* and trypanosome infection [1], [2], [31]. Our experimental *G. pallidipes* come from a virus-infected colony and no virus-free flies were available to test. SGH virus has also been reported at low levels in wild *G. pallidipes* (reviewed by [24]).

Natural SG infection rates of *G. pallidipes* with *T. brucei* are typically very low (<0.3%, [32]), and no SG infections were detected by dissection in recent surveys in Kenya [33] and Tanzania [34]. Since a strong MG immune response by itself is sufficient to block transmission of *T. brucei*, without any need for deployment of further immune defences in the foregut and SG, there is no inconsistency between our laboratory results and the observed refractoriness of *G. pallidipes* to trypanosome infection in the field. However, in laboratory *G. pallidipes*, the decreased ability to block SG colonisation makes *G. pallidipes* a very useful experimental fly for transmission of *T. brucei*.

## Materials and Methods

### Tsetse flies and trypanosomes

Tsetse flies were kept at 25°C and 70% relative humidity and fed on sterile defibrinated horse blood via a silicone membrane. Flies were given the infected bloodmeal for their first feed 24–48 hours post-eclosion, which consisted of cryopreserved bloodstream form trypanosomes of *T. b. brucei* J10 (MCRO/ZM/74/J10 [clone 1]) in defibrinated horse blood (approximately 10^6^ cells/ml). Infective bloodmeals were supplemented if necessary with final concentrations of 60 mM N-acetyl-glucosamine (NAG) [8] or 10 mM L-glutathione (GSH) [9] to increase infection rates. Results were usually combined from two replicate experiments to increase sample size, except for those shown in Tables 1 and 2 which were each derived from a single batch of flies.

### Spit samples

Spit samples were obtained from flies as described [13]; male and female flies were sampled on days 8–18 and 7–28 in two replicate experiments. Slides were fixed with 2% paraformaldehyde (PFA), washed three times with phosphate buffered saline (PBS) and then incubated with 1∶100× Hoechst 33258 DNA stain for 15 minutes. The slides were mounted using FluorSave reagent and viewed by fluorescence imaging to record the life cycle stage of the parasites using a DMRB microscope (Leica) equipped with a Colour Coolview camera (Photonic Science) and ImagePro Plus software (Media Cybernetics). Digital images of life cycle stages were quantified using Image J (http://rsb.info.nih.gov/ij/). Morphology and relative positions of the nucleus and kinetoplast were used to identify developmental stages [18], [19], [20]. Cells were assigned to the following developmental stages: long proventricular trypomastigote, asymmetrically dividing cell, short or long epimastigote, metacyclic.

### Dissection

Flies were killed by removing the head. Salivary glands were placed into a drop of PBS. Salivary gland hypertrophy (SGH) was recorded if the glands were grossly swollen; such glands also appear white rather than transparent. Whole tsetse alimentary tracts, from the proventriculus to the rectum, were placed into a separate drop of PBS. Infection of the proventriculus was examined in flies dissected 10–14 days after the infected feed; the proventriculus was cut from the MG immediately upon dissection and placed in a separate drop of PBS. Organs were viewed as wet mounts in PBS under bright field illumination (×100 magnification) and the presence of trypanosomes recorded.

### Statistics

The chi-squared test (Fisher's exact) was used for analysis of categorical data using http://www.graphpad.com/quickcalcs/contingency1.cfm. ANOVA was used for comparison of trypanosome cell types in spit samples from male and female flies. Numbers of trypanosomes were square-root transformed prior to analysis to normalise variances. The rate at which flies became positive for trypanosomes in spit samples was analysed by Kaplan Meier survival followed by Breslow (generalized Wilcoxon) testing. ANOVA and Kaplan-Meier data were processed using the statistical package SPSS version 18.0.
